# Preference for Male Risk Takers Varies with Relationship Context and Health Status but not COVID Risk

**DOI:** 10.1007/s40806-023-00354-3

**Published:** 2023-02-22

**Authors:** Cyril C. Grueter, Hannah Goodman, Nicolas Fay, Bradley Walker, David Coall

**Affiliations:** 1 Department of Anatomy, Physiology and Human Biology, School of Human Sciences, The University of Western Australia, M309, LB 5005, Perth, WA 6001 Australia; 2 Centre for Evolutionary Biology, School of Biological Sciences, The University of Western Australia, Perth, WA 6001 Australia; 3 School of Psychological Science, The University of Western Australia, Perth, WA 6001 Australia; 4 School of Medical and Health Sciences, Edith Cowan University, Joondalup, WA 6027 Australia

**Keywords:** Risk taking, Sexual selection, Mate choice, Health, COVID

## Abstract

Risk taking is more commonly shown by males than females and has a signalling function, serving to advertise one’s intrinsic quality to prospective mates. Previous research has established that male risk takers are judged as more attractive for short-term flings than long-term relationships, but the environmental and socioeconomic context surrounding female preferences for male risk takers has been overlooked. Using a survey instrument, we examined female preferences for male risk takers across 1304 females from 47 countries. We found preferences for physical risk takers to be more pronounced in females with a bisexual orientation and females who scored high on risk proneness. Self-reported health was positively associated with preferences for high risk takers as short-term mates, but the effect was moderated by country-level health, i.e. the association was stronger in countries with poorer health. The security provided by better health and access to health care may allow females to capitalise on the genetic quality afforded by selecting a risk-prone male whilst concurrently buffering the potential costs associated with the risk taker’s lower paternal investment. The risk of contracting COVID-19 did not predict avoidance of risk takers, perhaps because this environmental cue is too novel to have moulded our behavioural preferences.

**Supplementary Information:**

The online version contains supplementary material available at 10.1007/s40806-023-00354-3.

## Introduction

Risk taking is a sexually dimorphic behaviour, with males exhibiting a stronger innate inclination to be risk prone than females (Byrnes et al., 1999). Risk taking can be seen in multiple domains such as gambling, binge drinking, extreme sports, unprotected sex, reckless driving and drug use (e.g. Greitemeyer et al., 2013). Risk evaluation is also sex differentiated, with males perceiving potentially dangerous situations as less risky than females (Harris & Miller, 2000).

Sexual selection provides an explanatory framework for the androcentricity of risk taking. One pillar of sexual selection theory, intersexual selection or female choice, is concerned with the evolution of traits that make males more sexually attractive to females (Darwin, 1871). Akin to physical ornaments (e.g. testosterone-dependent masculine features), risk taking has the potential to convey honest/reliable information on the intrinsic quality of the performer of the risky activity (Kelly & Dunbar, 2001; Pawlowski et al., 2008; Wilke et al., 2006). Ultimately, non-lethal forms of risk taking can enhance a performer’s prestige and confer reproductive benefits (e.g. Chagnon, 1988). The signalling/sexual advertisement function of risk taking is supported by studies showing that males are more likely to engage in risky behaviours in the presence of females (McAlvanah, 2009; Pawlowski et al., 2008; but see Goodman, Grueter and Coall, in prep.) and especially in the presence of attractive females (Ronay & Hippel, 2010; see also Baker & Maner, 2008).

Risk taking as a demonstration of intrinsic quality is likely to be of particular importance in the context of choosing a short-term mate. In the pursuit of long-term partnerships, however, signals of parental investment should take precedence. Sexual strategies theory emphasises the fact that partner preferences vary with relationship type (Buss & Schmitt, 1993, 2019). For casual or short-term sexual liaisons, women desire strong, powerful and courageous men (or ‘cads’) who can provide them with genetic qualities that are likely to be passed on to offspring (Gangestad & Simpson, 2000). Traits indicative of the genetic quality that are prioritized when choosing a short-term mate include physical attractiveness (Kenrick et al., 1993) and a masculine physique (Little et al., 2007). Conversely, for long-term relationships, women place greater value on industriousness, relationship commitment and resource potential (Buss, 1989; Buss & Schmitt, 1993, 2019; Li et al., 2002).

The importance of relationship type as a determinant of preferences for risk taking has been empirically studied (Apalkova et al., 2018; Kelly & Dunbar, 2001; Sylwester & Pawłowski, 2011; see also Bassett & Moss, 2004). Sylwester and Pawłowski (2011) found that for short-term relationships both men and women preferred risk takers over risk avoiders. However, in the context of long-term relationships, risk avoidance was considered to be a favourable trait. Apalkova et al. (2018) showed that occasional (but not high) risk takers were judged highest on short-term attractiveness whereas low risk takers received the highest ratings on long-term attractiveness and provisioning quality. However, preferences for risk takers are also expected to be moderated by ecological and socioeconomic conditions.

We are not aware of any studies attempting to link preferences for risk takers to environmental context. There are, however, studies on how preferences for (anatomical) masculinity vary as a function of environmental context. Male facial masculinity is regarded as costly, and therefore an honest indicator of quality, with masculinity reflecting strength (Fink et al., 2007; Sell et al., 2009), long-term health and immunocompetence (Foo et al., 2017; Rhodes et al., 2003; Thornhill & Gangestad, 2006), and the prospects of heritable benefits (Gangestad & Simpson, 2000; Thornhill & Gangestad, 1996; but see Scott et al., 2013; Zaidi et al., 2019). More masculine men are, however, also perceived as less committed, less paternally invested and more philandering (Boothroyd et al., 2008; Kruger, 2006), and indeed do tend to display these traits (Polo et al., 2019; Rhodes et al., 2005). Women’s preferences for masculine versus feminine men are thus sensitive to trade-offs between the benefits (e.g. greater offspring fitness) and costs (e.g. low investment) associated with choosing a masculine mate (DeBruine et al., 2010; Gangestad & Simpson, 2000). Similar to physical masculinity, risk taking can be conceptualised as a behavioural manifestation of masculinity, with greater risk takers embodying traits associated with more masculine men. For example, Fessler et al. (2014) showed that males taking voluntary physical risks were perceived to be more formidable compared to risk-averse men.

In support of the putative link between masculinity and health/genetic quality, DeBruine et al. (2010) reported that female residents of countries characterised by poorer health (i.e. higher mortality rates and incidence of communicable disease and lower life expectancy) exhibited stronger preferences for male facial masculinity (see also DeBruine et al., 2011). Similarly, experimental work has shown that exposure to cues of pathogens results in stronger preferences for facial masculinity (Little et al., 2011). In environments teeming with potentially life-threatening health risks and poor healthcare, women are expected to derive an indirect fitness benefit from exerting stronger preferences for masculinity. However, McIntosh et al. (2017) failed to find support for the prediction that women prefer facial masculinity following exposure to pathogenic cues (see also Tybur et al., 2022). Additionally, two recent experimental studies have documented either a reduction in the preference for masculinised faces (Saribay et al., 2021) or a preference for feminine male faces under pathogen threat (Pereira et al., 2020). Moreover, in a cross-national survey, it was shown that women’s preferences for facial masculinity were *weaker* in countries with lower health and development indices (Marcinkowska et al., 2019).

Variables related to wealth/resource abundance and economic development may also covary with masculinity and–by inference–risk preferences. Marcinkowska et al. (2019) reported that women’s preferences for facial masculinity were stronger in countries with higher indices of human economic and social development. Scott et al. (2014) also found, using cross-cultural data, that preferences for masculinity were positively correlated with the Human Development Index, a country-level measure of social and economic development. If risk taking signals quality at the cost of low paternal investment, then risk taking should not be preferred in poor/resource-scarce environments. This is because only in wealthy environments with abundant resources and high levels of material security do women have the ‘freedom’ to neglect concerns about investment and select high-quality mates. By contrast, in resource-scarce environments, women will place a premium on investing partners, without regard for mate quality (Little et al., 2013; but see Lu et al., 2015). Alternatively, if risk taking signals formidability, then risk taking may be preferred in resource-scarce environments. Since resource-scarce environments are characterised by higher male competition (Daly & Wilson, 2001), females may obtain direct benefits from preferring risk takers, such as protection and resource provisioning (Kelly & Dunbar, 2001). The same has been proposed for physical masculinity (Puts, 2010; Saribay et al., 2021; Sell et al., 2009). Little et al. (2013) showed that after exposure to images of physical male-male competition and violence, women exhibited visual preferences in favour of male faces that signal physical strength and dominance. There is also some evidence from cross-cultural analyses that masculinity is a preferred trait under conditions of high male-male competition, as indexed by homicide rates and income inequality (Brooks et al., 2011; but see Marcinkowska et al., 2019).

The main aims of the present study are to synthesise the literature on risk taking (and attractiveness thereof) with the literature on masculinity preferences and test whether preferences for male risk takers as mates are moderated by relationship context and measures of health and development. Drawing on findings on masculinity preferences and sexual strategies theory, we derived the following predictions: (1) Male risk takers are preferred as short-term mates in low health, high-mortality environments (indexed by national life expectancy, self-reported health, COVID-19 cases); although an explanation is not explicitly tested in this study, this is likely because in such environments the benefit of selecting a risk taker (enhanced survival of offspring bearing this mate’s good genes) outweighs the potential costs of reduced paternal investment (*sensu* DeBruine et al., 2010; DeBruine et al., 2011). 2) Male risk takers are preferred as short-term mates in more developed/wealthier environments (index by individual-level income and country-level economic development); although an explanation is not explicitly tested in this study, this is likely because in such environments, assurance of investment may be a less pressing issue than securing heritable genetic benefits (*sensu* Little et al., 2013). 3) Risk takers are preferred as short-term mates when inequality (and thus competition) is high (*sensu* Brooks et al., 2011). We did not expect male risk takers to be attractive as long-term mates under any of the above conditions. Next, In line with previous research demonstrating that sociosexually unrestricted women preferred more facially masculine men than more sociosexually restricted women (Marcinkowska et al., 2019; Waynforth et al., 2005), we tested if bisexual women are more likely to exhibit preferences for male risk takers as both short- and long-term mates. Lastly, in keeping with research showing positive assortment for risk taking (with couples exhibiting comparable risk attitudes Bacon et al., 2014; Farthing, 2005; Wilke et al., 2006), we predict that there is a concordance between self-reported risk-taking dispositions and preferences for physical risk takers as short- and long-term mates.

We focus on physical (as opposed to, e.g. financial) risk taking since physical risk taking captures fitness-revealing traits such as strength (Farthing, 2005) and constitutes a good indicator of the underlying genetic quality of a mate (Sylwester & Pawłowski, 2011). Instead of relying on a biased WEIRD (Western, Educated, Industrialized, Rich, Democratic) sample, as often done in studies on mate preferences, we used a global sample of participants from 47 countries. The present study also offers a methodological improvement over previous cross-cultural/cross-national studies on masculinity preferences in that we explicitly address the ecological fallacy of extrapolating from aggregate patterns to lower-level patterns (Kuppens & Pollet, 2014; Pollet et al., 2014). More specifically, we empirically evaluate if the patterns observed at the country level correspond to patterns observed at the individual level. Positive inter-level covariation would be suggestive of similar processes operating at the country and at the individual levels.

## Materials and Methods

### Data Collection

The questionnaire was created using Qualtrics and comprised three sections: participant information, male vignette and a comprehension test. The first section consisted of questions relating to the participants’ country of residence, age, sex, relationship status, sexual orientation, total household income per year and health. The second section consisted of a vignette describing a risk-seeking male and his occupation (e.g. *Pete works as a teacher. In his spare time, he likes to rock climb and abseil outdoors. He enjoys the adrenaline rush associated with these activities*). The participants were then asked to rate how attractive they found the male as a short- and long-term mate on a 5-point Likert scale (1: very unattractive, 2: somewhat unattractive, 3: neither unattractive nor attractive, 4: somewhat attractive and 5: very attractive). Participants also rated how much they enjoyed adrenaline-inducing activities, as liking or disliking these (physically risky) activities could impact how relatable, and thus how attractive, they found the male in the vignette. The final section listed three comprehension questions that were used to filter out participants who did not pay attention while completing the questionnaire.

With a 95% confidence interval and an effect size (Glass’ delta) of 0.478 (based on Sylwester & Pawłowski, 2011), the minimum sample size for questions pertaining to the attractiveness of short-term mates was 831 and the minimum sample size for questions pertaining to the attractiveness of long-term mates was 1247. Questionnaire participants were crowdsourced via the Amazon Mechanical Turk (MTurk) platform. There were three qualifying criteria for questionnaire respondents to be included in the study. The participant had to be female, aged between 18 and 40 and identify as heterosexual or bisexual. The reason for restricting recruitment to heterosexual and bisexual females was that the questionnaire was centered around the participant’s attraction to a male from the perspective of sexual selection and risk taking was regarded as an advertisement strategy for reproductive mates. The total questionnaire sample included 1304 participants (957 heterosexual and 347 bisexual females). Table S5 shows the countries of residence represented in the sample.

To test our predictions regarding women’s preferences for male risk takers, we sourced from public databases the following nation-level data: adult life expectancy in years (capturing population health; obtained from The World Health Organisation Database); Human Development Index HDI (a composite statistic (consisting of lifespan, education level and GDP per capita) which captures levels of national development, obtained from The United Nations Database); and the GINI coefficient (a measure of the distribution of income in a country that captures inequality, obtained from The World Bank Database). These variables have previously been used in cross-national analyses of women’s facial masculinity preferences (Marcinkowska et al., 2019). The impact of COVID-19 on the attractiveness of male risk taking was assessed through total COVID-19 cases per 1 million individuals in a given country (as of the week of the questionnaire response).

### Data Analysis

Data were analysed with cumulative link mixed models fitted with the Laplace approximation. Attractiveness of risk takers as either short- or long-term mates on a 5-point Likert scale was modelled as the dependent variable. The main fixed (or constant) effects consisted of both country-level and individual-level variables. The country-level variables were life expectancy (a continuous variable), HDI (continuous), GINI coefficient (continuous) and total COVID-19 cases per 1 million individuals (continuous; ln-transformed). The individual-level variables were age (continuous), income (continuous), self-reported health (on a 10-point Likert scale) and relationship status (a categorical variable comprised of ‘married/engaged’, ‘dating’ and ‘not in a relationship’). The model also contained the two-way, cross-level interaction between life expectancy and individual health. If the interaction effect was not statistically significant, it was removed from the model and the model was rerun without the interaction effect. To examine potential collinearity among the various fixed effects, we determined variance inflation factors (VIF) applied to a standard linear model without the random effects. Using a stringent cut-off of 2, the only variables that displayed collinearity and thus could not be placed in the same model were life expectancy and HDI. We, therefore, created two separate models, one focusing on health (the ‘health model’) and one focusing on development (the ‘development model’). Only responses from heterosexual females were used in these models. To incorporate the dependency among responses from the same country, we included the name of the participant’s country of residence as a random (or varying) effect. Geographical region was included in the models as a second random effect because of the possible non-independence between countries with a similar geographical location (e.g. similar climate, cultural history) (Marcinkowska et al., 2019). We used the 19 UN geographical regions for statistical use (http://unstats.un.org/unsd/methods/m49/m49regin.htm). We also included the random slopes for the effects of age, income, health and relationship status on attractiveness ratings to allow these variables to vary between levels of the factor ‘country’. This was done to avoid inflating type 1 error rates (Schielzeth & Forstmeier, 2009). Before fitting the model, we z-transformed all covariates to a mean of zero and a standard deviation of one to achieve comparable estimates and to increase the likelihood of model convergence (Schielzeth, 2010). The models had the following structure:

- Model with life expectancy (‘Health model’): *clmm(Attractiveness of risk taker* ~ *life expectancy* + *Gini* + *age* + *income* + *health* + *relationship status* + *COVID-19 cases* + *life expectancy:health* + *(1|country)* + *(1|region)* + *(0* + *age|country)* + *(0* + *income|country)* + *(0* + *health|country)* + *(0* + *relationship status|Country)*
- Model with HDI (‘Development model’): *clmm(Attractiveness of risk taker* ~ *HDI* + *Gini* + *age* + *income* + *health* + *relationship status* + *COVID-19 cases* + *HDI:income* + *(1|country)* + *(1|region)* + *(0* + *age|country)* + *(0* + *income|country)* + *(0* + *health|country)* + *(0* + *relationship status|country)*

We also ran two additional models, one examining the effect of sexual orientation (bisexual vs. heterosexual) on female preferences for male risk takers (the ‘sociosexual model’) and one assessing the association between self-reported risk attitude and preferences for male risk takers (the ‘assortativity model’). In both models, a number of control variables were incorporated as well, viz*.* life expectancy, age, health and relationship status.

Model fitting was done in R version 3.6.0 (R Core Development Team, 2019) using the function ‘clmm’ of the package ordinal (Christensen, 2013). To calculate VIF, we used the function ‘vif’ of the R package car (Fox & Weisberg, 2011).

## Results

### Health and Development Models

**Fig. 1:**
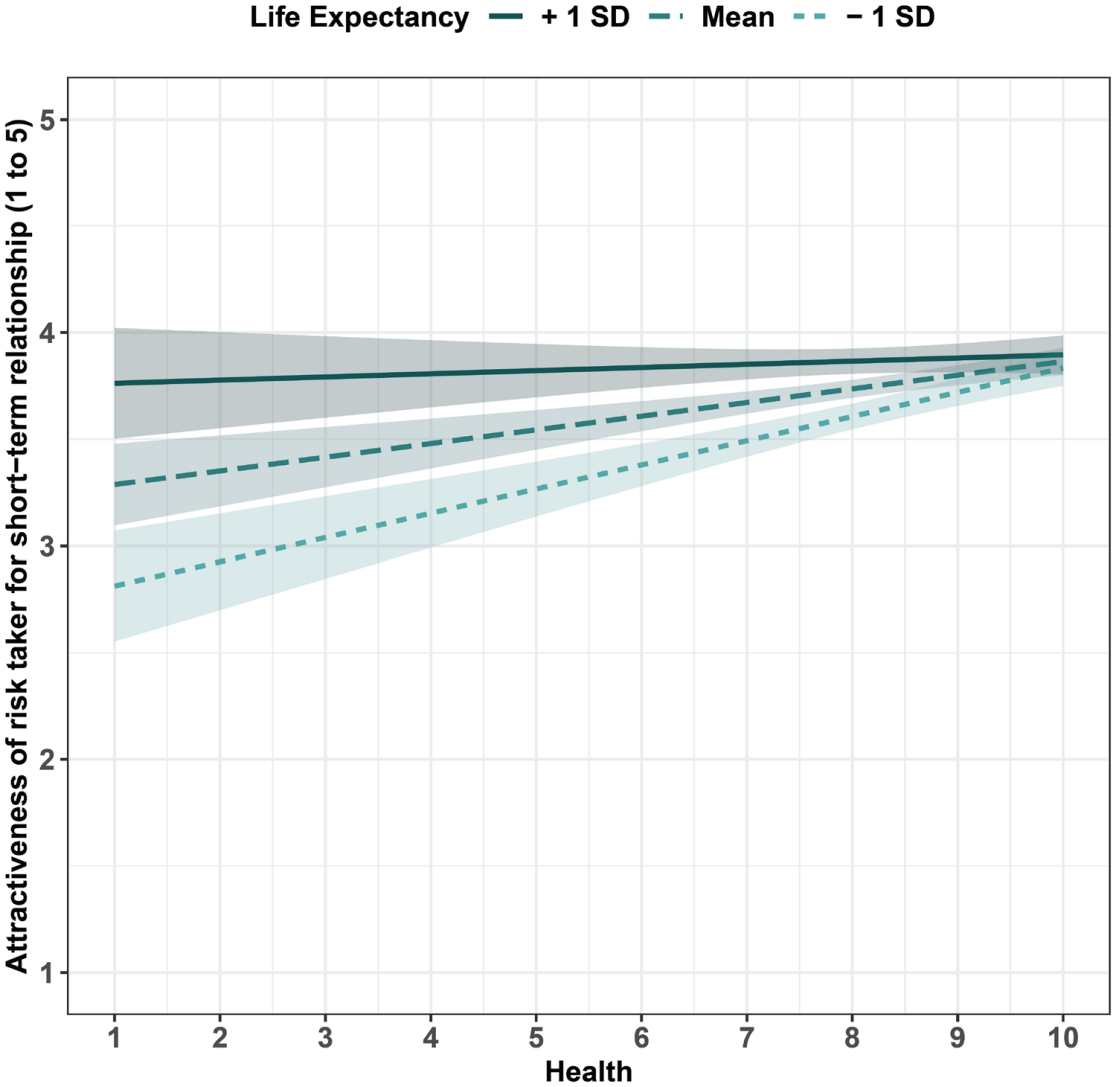
Relationship between attraction to male risk takers as short-term mates (on a 1–5 Likert scale) and self-reported health (on a 1–10 Likert scale) as moderated by country-level life expectancy (+1 standard deviation, mean and −1 standard deviation)

The fixed effect of country-level life expectancy indicates that heterosexual females from countries with a higher life expectancy found high risk-taking males more attractive as short-term mates. The fixed effect of individual-level health indicates that heterosexual females with higher self-reported health were more attracted to high risk-taking males. The interaction between the fixed effects indicates that the influence of individual-level health is moderated by country-level life expectancy: the positive association between individual-level health and attraction to high risk-taking males is stronger among heterosexual females from countries with a lower life expectancy (Fig. 1). No other variables were associated with an attraction to male risk takers, including contracting COVID-19 (Table 1). Individual-level health did not show any noticeable association with preferences for risk takers as long-term mates; there was, however, a weak effect of life expectancy, albeit in the opposite direction as was reported for short-term mates (Table S1). The models with HDI (‘development models’) did not reveal any significant association with preferences for either type of risk takers (Tables 2 and S2).

**Table 1:** The health model for the attractiveness of risk takers as short-term mates

	Estimate	Standard error	*z*	*p*
Country-level life expectancy	0.216	0.086	2.501	0.012
Gini	0.093	0.088	1.051	0.294
Age	−0.005	0.065	−0.070	0.944
Income	0.048	0.124	0.388	0.698
Individual-level health	0.201	0.061	3.251	0.001
Relationship status _Dating_^a^	0.712	0.607	1.172	0.241
Relationship status _Married_	0.499	0.665	0.750	0.453
Relationship status _Not in a relationship_	0.457	0.739	0.618	0.536
COVID-19	0.049	0.086	0.561	0.574
Country-level life expectancy * individual-level health	−0.183	0.057	−3.163	0.001

^a^The subscript refers to the level of a categorical predictor

**Table 2:** The development model for the attractiveness of risk takers as short-term mates

	Estimate	Standard error	*z*	*p*
HDI	0.048	0.111	0.439	0.660
Gini	0.110	0.095	1.158	0.246
Age	−0.005	0.065	−0.081	0.935
Income	0.074	0.128	0.581	0.561
Health	0.174	0.095	1.832	0.066
Relationship status _Dating_	0.933	0.624	1.495	0.135
Relationship status _Married_	0.756	0.698	1.082	0.279
Relationship status _Not in a relationship_	0.794	0.741	1.072	0.283
COVID-19	0.065	0.097	0.667	0.504

### Sociosexual Model

This model tested if sexually more open (bisexual) women differ from heterosexual women in terms of their preferences for male risk takers. Bisexual females were more likely to exhibit preferences for male high risk takers, both in a short-term and long-term relationship contexts (Tables 3 and S3). This model also revealed a strong association between the life expectancy-health interaction and attractiveness of risk takers as short-term mates; this finding is consistent with the health model detailed above.

**Table 3:** The sociosexual model for the attractiveness of risk takers as short-term mates

	Estimate	Standard error	*z*	*p*
Life expectancy	0.199	0.053	3.747	0.0001
Health	1.667	0.505	3.301	0.0009
Age	−0.009	0.012	−0.793	0.427
Relationship status _Dating_	0.730	0.659	1.107	0.268
Relationship status _Married_	0.717	0.658	1.090	0.275
Relationship status _Not in a relationship_	0.635	0.662	0.959	0.337
Sexual orientation _Heterosexual_	−0.474	0.155	−3.051	0.002
Country-level life expectancy * individual-level health	−0.019	0.006	−3.047	0.002

### Assortativity Model

**Fig. 2:**
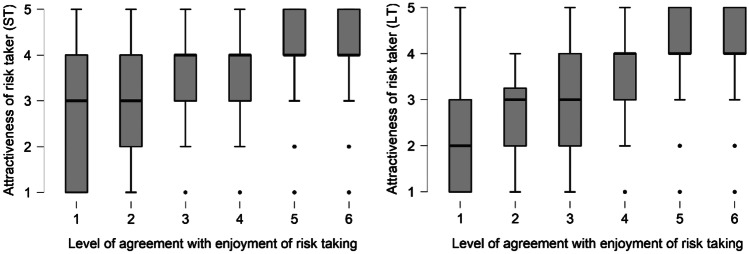
Self-reported risk attitude vs. attractiveness of high risk takers as short-term (ST) and long-term (LT) mates. Attractiveness was scored using a 1–5 Likert scale. The level of agreement with a statement about the enjoyment of physical risk taking ranges from 1 (strongly disagree) to 6 (strongly agree)

Participants’ self-reported level of agreement with a statement about the enjoyment of physical risk taking (‘I enjoy physical activities that give an adrenaline rush’) was a strong predictor of how attractive they rated high risk takers as both short-term (Table 4; Fig. 2) and long-term mates (Table S4). There was also a strong association between the life expectancy-health interaction and attractiveness of risk takers as short-term mates, a finding that is in line with the health model.

**Table 4:** The assortativity model for the attractiveness of risk takers as short-term mates

	Estimate	Standard error	*z*	*p*
Adrenaline _Disagree_	−1.685	0.304	−5.530	3.20e-08
Adrenaline _Somewhat agree_	−0.376	0.166	−2.261	0.023
Adrenaline _Somewhat disagree_	−1.022	0.269	−3.794	0.0001
Adrenaline _Strongly agree_	0.405	0.209	1.937	0.052
Adrenaline _Strongly disagree_	−2.724	0.356	−7.641	2.16e-14
Life expectancy	0.269	0.075	3.583	0.0003
Age	0.080	0.071	1.131	0.258
Health	0.078	0.067	1.163	0.244
Relationship status _Dating_	−0.130	0.991	−0.131	0.895
Relationship status _Married_	−0.286	0.992	−0.289	0.772
Relationship status _Not in a relationship_	−0.120	0.993	−0.121	0.903
Country-level life expectancy * individual-level health	−0.207	0.059	−3.494	0.0004

## Discussion

Drawing on a sample of 1304 females from 47 countries, we show that self-reported health was strongly associated with female preferences for male high risk takers as short-term mates. However, the positive association between self-reported health and attractiveness of male risk takers was weaker in countries with higher health. The generally positive relationship between health-related variables and attraction to male risk takers echoes recent research on masculinity preferences which were more strongly expressed in countries with higher national health indices (Marcinkowska et al., 2019). They do not corroborate the prediction that in areas with elevated health risks and poorer health outcomes, women prioritize the indirect fitness benefit they obtain from exerting preferences for masculinity (DeBruine et al., 2010). One reason why men flaunting masculinity were generally unattractive to females in a high pathogen environment may lie in the fact that these males may be the most immunocompromised (as a result of the immunosuppressive effect of testosterone; Foo et al., 2017) and are thus unable to realize their full biological potential. The reason for the positive effect of health-related variables on risk taker preferences may be that the security afforded by better health and access to health care makes paternal investment less important and allows women to exhibit preferences in favour of males with behavioural cues indicating genetic quality (at the expense of investment). A potential complementary explanation could be that women in healthier countries have greater control over whether they become pregnant in a short-term relationship (through contraceptives and abortion) and therefore can afford to choose a risk-prone male partner. Variables pertaining to development and wealth (individual-level income and country-level economic development) and inequality/competition carried no significant explanatory value in our statistical models.

Our results demonstrate that relationship context matters when it comes to preferences for risk takers. The above associations between respondent health and reported attractiveness of risk takers were seen only in the context of short-term relationships where the potential benefits associated with genetic qualities outweigh the costs associated with risk taking (in terms of reduced paternal investment). In long-term partnerships, the disadvantages of being attracted to a habitual risk taker (lack of paternal support as a result of indifference or mortality) likely become excessive and thus curb attraction to risk takers.

We also found that bisexual women are more likely than heterosexual women to exhibit preferences for male risk takers as both short- and long-term mates. This could indicate that bisexual participants have less conservative perceptions surrounding mate attraction, relationships and male paternal investment. Similar findings have been reported for masculinity preferences, with sociosexually unrestricted women preferring more facially masculine men than more sociosexually restricted women (Marcinkowska et al., 2019; Waynforth et al., 2005).

One variable that showed a strong association with preferences for high risk takers, even after controlling for health and demographic variables, was the respondent’s self-reported risk taking tendencies. Positive assortative matching, whereby mates resemble each other in a variety of characteristics such as age, education, wealth, status and physical appearance (Buston & Emlen, 2003; Watson et al., 2004), has been reported for a number of domains. Our findings that assortativity extends to risk taking are in line with a number of other studies (Bacon et al., 2014; Cobey et al., 2013; Farthing, 2005; Wilke et al., 2006). This positive assortment for risk taking may ultimately benefit couples in terms of greater relationship satisfaction and stability (Weisfeld et al., 1992) and enhanced reproductive success (Bereczkei & Csanaky, 1996). However, our study measured stated preferences only, which do not always predict actual choices (Todd et al., 2007).

The risk of contracting COVID-19 did not predict the avoidance of risk takers. One explanation may be that this environmental cue is too novel to have moulded our behavioural preferences. Alternatively, it is conceivable that respondents in all sampled countries were similarly exposed to the pandemic via the media, which may have affected their judgments and perceptions more than local or first-hand exposure with COVID-19. A recent study examining women’s preference for male facial masculinity also found that concern about contracting COVID-19 did not influence preference ratings (Pazhoohi et al., 2021).

There are some potential limitations inherent in the study design that are worth mentioning. One assumption of our study is that male risk taking represents a quality signalling device (Kelly & Dunbar, 2001; Pawlowski et al., 2008; Wilke et al., 2006). This assumption is anchored in sexual selection theory and supported by indirect empirical evidence (McAlvanah, 2009; Pawlowski et al., 2008; Ronay & Hippel, 2010). However, future studies could attempt to generate more direct evidence for a functional link between male physical risk taking and quality indicators such as strength and immunocompetence. Another potential limitation concerns the self-reported health variable which can fall victim to the ‘reference group effect’ (Heine et al., 2002). A self-reported health rating of ‘4’ in one country might be a ‘6’ in another, due to cultural differences. This could be avoided in future by providing a descriptive standard that participants can compare themselves to (Heine et al., 2002). Lastly, while our sample was global in that it included participants from 47 countries across all inhabited continents, almost half of the sample (570 participants) came from Canada, the UK, the USA and Australia. Twenty-two of the countries only had a single participant, and eight countries only had two participants. This imbalance is inevitable as MTurk is less commonly used in non-WEIRD countries.

Several of our predictions about the attractiveness of male risk takers were derived from the literature on masculinity preferences (DeBruine et al., 2010; Marcinkowska et al., 2019; Saribay et al., 2021). Future studies could take this research to another level by investigating interactions between masculinity and risk taking. To determine whether masculinity influences the attractiveness of risk taking, one could ask females to rate the attractiveness of male risk taking vignettes that are accompanied by visual depictions of varying masculinity. Another aspect of future research revolves around the possibility that risk preferences are affected by individual variation in women’s endocrine profiles. It has been argued that women exhibit stronger preferences for indicators of biological quality (such as risk taking) during the peri-ovulatory phase of the menstrual cycle (Gangestad & Thornhill, 2008; but see Jones et al., 2018) when they can secure indirect fitness benefits for their offspring. Without the collection of endocrine data from study participants, the possibility of a fecundability effect cannot be ruled out. Lastly, in future studies, the quantification of self-reported risk proneness used in this study (i.e. level of agreement with a statement about the enjoyment of adrenaline-inducing activities) could be replaced with a validated risk-taking scale, such as the one developed by Wilke et al. (2014).

In sum, our findings show that under more favourable health conditions, women exhibit preferences for risk takers as short-term mates, but this association does not carry on to long-term mates. The security afforded by better health and access to health care may reduce the pressure on women to favour parental investment and instead allow them to prioritise the putative genetic quality of a risk prone male. Moreover, we found preferences for risk takers to be more pronounced in individuals with a bisexual orientation and individuals who themselves score high on risk proneness.

## Supplementary Information

Below is the link to the electronic supplementary material.

Supplementary file1 (DOCX 20 KB)

## Data Availability

Data are available from the Dryad Digital Repository: https://doi.org/10.5061/dryad.tx95x6b0r.
