# Supplementary material for: Preference for Male Risk Takers Varies with Relationship Context and Health Status but not COVID Risk
Source: Evol Psychol Sci. 2023 Feb 22:1–10. Online ahead of print. doi: 10.1007/s40806-023-00354-3 (PMC9944803; doi:10.1007/s40806-023-00354-3)
Supplement: Supplementary file 1 — Supplementary file1 (DOCX 20 KB) [file 40806_2023_354_MOESM1_ESM.docx]

**Supplementary Information**

**Table S1**. The health model for the attractiveness of risk takers as long-term mates.

|  | Estimate | Standard error | z | p |
| --- | --- | --- | --- | --- |
| Life expectancy | -0.188 | 0.084 | -2.222 | 0.026 |
| Gini | -0.069 | 0.086 | -0.798 | 0.425 |
| Age | -0.061 | 0.065 | -0.936 | 0.349 |
| Income | 0.026 | 0.067 | 0.391 | 0.695 |
| Health | 0.075 | 0.071 | 1.051 | 0.293 |
| Relationship status _Dating_ | 0.877 | 0.946 | 0.927 | 0.354 |
| Relationship status _Married_ | 0.639 | 0.960 | 0.665 | 0.505 |
| Relationship status _Not in a relationship_ | 0.876 | 0.948 | 0.924 | 0.355 |
| COVID-19 | 0.165 | 0.089 | 1.847 | 0.064 |

**Table S2**. The development model for the attractiveness of risk takers as long-term mates.

|  | Estimate | Standard error | z | p |
| --- | --- | --- | --- | --- |
| HDI | -0.174 | 0.103 | -1.690 | 0.091 |
| Gini | -0.107 | 0.106 | -1.003 | 0.316 |
| Age | -0.058 | 0.064 | -0.895 | 0.371 |
| Income | 0.029 | 0.069 | 0.419 | 0.675 |
| Health | 0.077 | 0.070 | 1.096 | 0.273 |
| Relationship status _Dating_ | 0.931 | 0.940 | 0.991 | 0.321 |
| Relationship status _Married_ | 0.691 | 0.956 | 0.723 | 0.469 |
| Relationship status _Not in a relationship_ | 0.933 | 0.938 | 0.994 | 0.320 |
| COVID-19 | 0.159 | 0.093 | 1.700 | 0.089 |

**Table S3**. The sociosexual model for the attractiveness of risk takers as long-term mates.

|  | Estimate | Standard error | z | p |
| --- | --- | --- | --- | --- |
| Life expectancy | -0.148 | 0.040 | -3.656 | 0.0002 |
| Health | -0.869 | 0.361 | -2.406 | 0.016 |
| Age | -0.004 | 0.011 | -0.435 | 0.663 |
| Relationship status _Dating_ | 0.413 | 0.742 | 0.556 | 0.577 |
| Relationship status _Married_ | 0.284 | 0.740 | 0.384 | 0.701 |
| Relationship status _Not in a relationship_ | 0.245 | 0.744 | 0.329 | 0.741 |
| Sexual orientation _Heterosexual_ | -0.322 | 0.141 | -2.272 | 0.023 |
| Life expectancy * health | 0.011 | 0.004 | 2.504 | 0.012 |

**Table S4**. The assortativity model for the attractiveness of risk takers as long-term mates.

|  | Estimate | Standard error | z | P |
| --- | --- | --- | --- | --- |
| Adrenaline _Disagree_ | -2.560 | 0.304 | -8.411 | < 2e-16 |
| Adrenaline _Somewhat agree_ | -0.608 | 0.168 | -3.613 | 0.0003 |
| Adrenaline _Somewhat disagree_ | -1.874 | 0.262 | -7.143 | 9.12e-13 |
| Adrenaline _Strongly agree_ | 0.825 | 0.211 | 3.901 | 9.60e-05 |
| Adrenaline _Strongly disagree_ | -3.310 | 0.356 | -9.299 | < 2e-16 |
| Life expectancy | 0.018 | 0.066 | 0.274 | 0.784 |
| Age | 0.017 | 0.070 | 0.250 | 0.802 |
| Health | -0.099 | 0.067 | -1.486 | 0.137 |
| Relationship status _Dating_ | 0.693 | 1.064 | 0.652 | 0.514 |
| Relationship status _Married_ | 0.590 | 1.064 | 0.555 | 0.579 |
| Relationship status _Not_in_a_relationship_ | 0.800 | 1.065 | 0.751 | 0.452 |
| Life expectancy * health | 0.012 | 0.057 | 0.216 | 0.828 |

**Table S5.** The countries of residence of the questionnaire participants.

| **Country of residence** | **Number of participants** |
| --- | --- |
| United States | 407 |
| Brazil | 112 |
| India | 101 |
| Italy | 80 |
| United Kingdom | 76 |
| Canada | 71 |
| Australia | 16 |
| Spain | 14 |
| Germany | 7 |
| Venezuela | 7 |
| Mexico | 5 |
| France | 4 |
| Ireland | 4 |
| Jamaica | 4 |
| Nigeria | 3 |
| Philippines | 3 |
| Singapore | 3 |
| Algeria | 2 |
| Argentina | 2 |
| Finland | 2 |
| Kenya | 2 |
| Netherlands | 2 |
| North Macedonia | 2 |
| Romania | 2 |
| South Arica | 2 |
| Bahrain | 1 |
| Bangladesh | 1 |
| Belarus | 1 |
| Botswana | 1 |
| Bulgaria | 1 |
| China | 1 |
| Colombia | 1 |
| Czech Republic | 1 |
| Ethiopia | 1 |
| Israel | 1 |
| Jordan | 1 |
| Kuwait | 1 |
| Poland | 1 |
| Portugal | 1 |
| Russia | 1 |
| Rwanda | 1 |
| Saint Vincent and the Grenadines | 1 |
| Serbia | 1 |
| Trinidad and Tobago | 1 |
| Turkey | 1 |
| Ukraine | 1 |
| Uruguay | 1 |
